# Harman Measurements for Thermoelectric Materials and Modules under Non-Adiabatic Conditions

**DOI:** 10.1038/srep39131

**Published:** 2016-12-14

**Authors:** Im-Jun Roh, Yun Goo Lee, Min-Su Kang, Jae-Uk Lee, Seung-Hyub Baek, Seong Keun Kim, Byeong-Kwon Ju, Dow-Bin Hyun, Jin-Sang Kim, Beomjin Kwon

**Affiliations:** 1 Center for Electronic Materials, Korea Institute of Science and Technology (KIST), Seoul, 02792, Republic of Korea; 2 Display and Nanosystem Laboratory, College of Engineering, Korea University, Seoul, 02841, Republic of Korea; 3 Department of Nanomaterials Science and Technology, Korea University of Science and Technology, Daejeon, 34113, Republic of Korea

## Abstract

Accuracy of the Harman measurement largely depends on the heat transfer between the sample and its surroundings, so-called parasitic thermal effects (PTEs). Similar to the material evaluations, measuring thermoelectric modules (TEMs) is also affected by the PTEs especially when measuring under atmospheric condition. Here, we study the correction methods for the Harman measurements with systematically varied samples (both bulk materials and TEMs) at various conditions. Among several PTEs, the heat transfer via electric wires is critical. Thus, we estimate the thermal conductance of the electric wires, and correct the measured properties for a certain sample shape and measuring temperature. The PTEs are responsible for the underestimation of the TEM properties especially under atmospheric conditions (10–35%). This study will be useful to accurately characterize the thermoelectric properties of materials and modules.

The Harman method assesses the thermoelectric (TE) figure-of-merit, *Z* = *α*^2^/*ρk* of a material or a TEM simply based on its voltage responses to an alternating current (AC) and a direct current (DC), where *α* is the Seebeck coefficient, *ρ* is the electrical resistivity, *k* is the thermal conductivity, and *T* is the absolute temperature^1^. Unlike the separate measurements^2^ of *α*, *ρ*, and *k*, the Harman method requires a single apparatus and a single sample preparation, hence essentially involves smaller uncertainties in the measurements^3^. However, the accuracy of the Harman method has often been questioned, as it is highly sensitive to the heat transfer from or to the sample and the electrical contact resistance^3^,^4^,^5^,^6^,^7^,^8^,^9^. Without correcting the parasitic thermal effects (PTEs) in the Harman method, *Z* measured for a single material could vary more than 50% among the samples with different dimensions or different electrical contact configurations^3^,^6^,^8^.

At any temperature, heat transfer significantly affects the Harman measurement. Even with thin electric wires (diameters of few tens of μm), the heat flows via the wires reduce the temperature gradient across the specimen as well as DC voltage response^3^,^6^,^8^. Simply reducing the thermal conductance of the electric wires would not completely solve the problem, as it mostly leads to the increases of the electrical resistance and also Joule heating of the wires. Radiative heat transfer considerably increases at high temperature, if the sample and the surrounding are at different temperatures^3^,^4^,^7^,^9^. Thus, the use of radiation shield is helpful to lower the error due to the radiation below few percentages^4^,^7^. Convective heat transfer also disturbs the temperature distribution within the sample, and can cause an unpredictable impact.

There have been various efforts to correct the PTEs involved in the Harman method^3^,^4^,^6^,^8^,^9^,^10^. Heat transfer models that account for the TE effects and the PTEs were presented^3^,^4^,^6^,^8^,^9^,^10^. Models predict that a sample with smaller thermal conductance, i.e. a sample with longer and narrower shape, is subject to larger heat flow through the electric wires, which, in turn, results in larger underestimation of *Z*^3^,^6^,^8^,^10^. To correct the PTEs, the dependence of error on the sample geometry was experimentally studied, and correction factors were estimated^6^. Another correcting scheme is to fit the measured AC and DC voltage responses to the theoretical model, which requires additional physical properties such as Seebeck coefficient or emissivity^3^,^8^,^10^.

Although the Harman method has been popularly used to evaluate *Z* of TEMs^11^,^12^, there has been much less attention to the influence of the PTEs on these measurements. As the TEMs use thick electric wires (diameters of few mm), the thermal loss would not be negligible. If the electric resistance (*R*) of the TEM is few tens of mΩ, the electrode resistance and contact resistance would also be important. In addition, the thermal loss in atmospheric condition is expected to be great regardless of the TEM sizes.

Here, we investigate the temperature-dependent PTEs on the Harman method both for the bulk materials and TEMs. We model the heat transfer involved in the Harman measurement, and fit to the extracted temperature gradient (Δ*T*) and measured *Z*. By measuring the samples with systematically varied sizes, the thermal properties related to PTEs and correction factors are uncovered at various temperatures.

## Sample Preparation

### Material samples

Test materials are Bi_2_Te_3−*x*_Se_*x*_ (n-type) and Bi_2−*x*_Sb_*x*_Te_3_ (p-type) fabricated via various processes such as hot extrusion, spark plasma sintering (SPS)^13^, and hot press^14^. As the TE properties such as *α*, *ρ*, and *k* of the materials all affect the PTEs^3^,^4^,^5^,^6^,^8^,^9^,^10^, testing several materials enables to see the link between the PTEs and TE properties, and helps to confirm the validity of the developed method. For various-temperature measurements (300~380 K), we especially employed three types of test materials fabricated by hot-extrusion method. Table 1 lists the physical properties at 300 K and geometries of these samples. Figure S1 (supplementary information) shows *α* and *ρ* measured over the entire temperature range. In addition to these materials, we also tested materials fabricated by SPS and hot-press technique (cross-section area of 2 × 1.6 mm^2^) for room-temperature measurements. To systematically assess the influence of electrical and PTEs on the Harman measurement, we prepared different sizes of samples. For each type of the test materials, we cut the sample to shorter length (*L*) while keeping the same area (*A*), and conducted further measurements.

### TEM samples

TEMs consist of multiple π-shaped thermocouples that are connected electrically in series and thermally in parallel. These thermocouples are sandwiched between two substrates. Table 2 summarizes the detail information for the TEMs. Figure S2 (supplementary information) shows *α* and *ρ* of the thermocouple materials measured over the entire temperature range. To vary the electrical and thermal resistance of TEMs, we fabricated four samples with different numbers of thermocouples (2, 4, 8, and 12) employing Cu/epoxy substrates. To study the substrate effect, we also prepared two other TEMs using Al_2_O_3_ and Si/SiO_2_ substrates which include 12 thermocouples.

## Overview of Harman Measurement

### Material measurement

A sample is suspended by two pairs of Cu wires in a 5-mm-thick quartz vacuum chamber (≤10^−4^ Torr). One pair of Cu wires serves voltage probes and another pair of wires supplies current (*I* = 25 mA) to the sample. The Cu wires are ~30 mm long with a diameter of 50 μm. Cu foils (thickness of ~500 μm) are attached at each sample end surface to ensure uniform current distribution. Measurement of *V*_*AC*_ provides the electrical resistivity (*ρ*_*t*_), and an additional measurement of *V*_*DC*_ gives TE figure-of-merit (*Z*). Details of the Harman measurement are available elsewhere^3^,^8^.

For the various-temperature measurements, a sample is positioned within a graphite radiation shield (*d* = 30 mm, *L* = 40 mm) that is again located within the vacuum chamber. The vacuum chamber is radially surrounded by six halogen lamps (*d* = 10 mm, *L* = 150 mm) with a radial gap distance of 20 mm. The halogen lamps are vertically centered with the radiation shield and are fixed by a surrounding insulation material. Temperature of the shield, *T*_*0*_, is measured by an inserted K-type thermocouple, and is controlled by a PID controller. To ensure the stability and uniformity of the temperature, we initiated all the measurements after maintaining the temperature over 30 minutes. We ramped the temperature from ~300 K to 380 K with an interval of ~30 K.

### TEM measurement

TEM measurements follow the same measuring protocol to the materials except the type of the electric wires. For a TEM, not the thin Cu wires but a pair of stranded lead wires (*d*~1 mm) are directly soldered to the TEM electrodes. As the Harman measurement requires four electrical terminals, the ends of the stranded wires are split to four pieces. For a small-size TEM, the electrical resistance of the stranded wires is not negligible. Thus, to eliminate the influences of the electric wire resistance (*R*_*wire*_), we first measure *R*_*wire*_, and subtract *IR*_*wire*_ from all the measured voltage data. For estimating the influence of the convective heat flow, we measured the TEMs under both an atmospheric and vacuum condition. For measuring a TEM above 100 °C, the melting point of the solder is critical. In-Sn, Bi-Sn, or other In based solders melt near 100 °C such that the TEMs made with such solders are not suitable for the high-temperature measurements.

## Theory

### Model for material measurement

**Figure 1:**
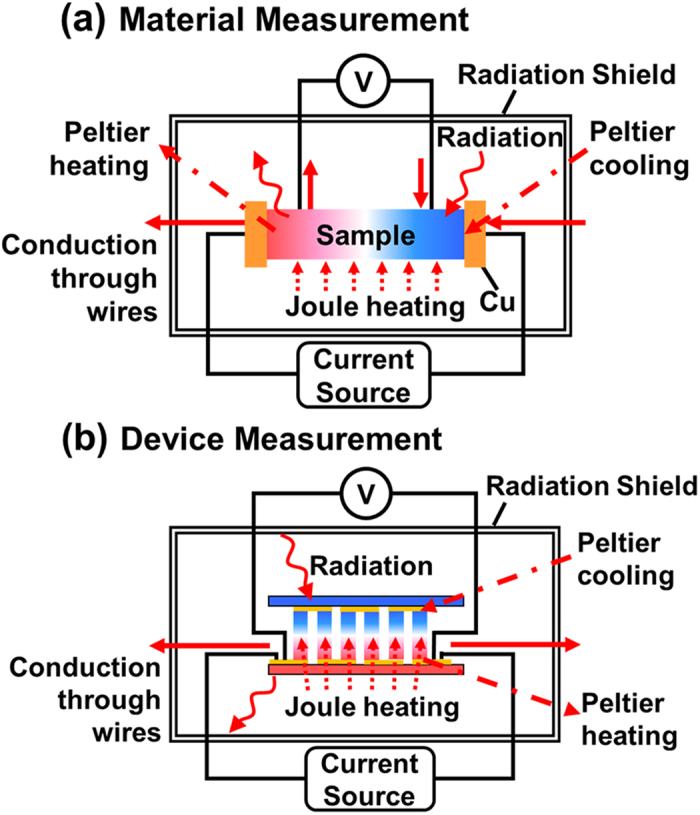
Schematics of the heat transfer models for (**a**) a material measurement, and (**b**) a module measurement.

Figure 1 shows the schematics for the heat transfer models. Heat transfer models consider the DC measurement configuration where Peltier effects, Joule heating within the sample, conduction via electric wires, and radiation from or to the sample are the dominant thermal effects. For a material measurement, the relation between the intrinsic *Z* (*Z*_*i*_) and the measured *Z* (*Z*_*m*_) is as below^8^:


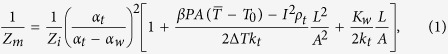


where *β* is the radiative heat transfer coefficient, *P* is sample perimeter, 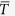 is an average temperature across the sample, and *K* is the thermal conductance. The subscripts *t* and *w* indicate the test material and electric wire, respectively. Although the square bracket contains a second order polynomial of *L*/*A*, 1/*Z*_*m*_ empirically is a linear function of *L*/*A*^3^,^6^,^8^, implying that Δ*T* could be a first or second order polynomial of *L*/*A*. Theoretically, Δ*T* is described by a hyperbolic function of *L* as shown in Fig. S3 (supplementary material), and has similar trend to the second order polynomial. Our observations of Δ*T* based on Δ*T* = (*V*_*dc*_−*V*_*ac*_)/(*α*_*t*_−*α*_*w*_) have also shown that Δ*T* fits well with the second order polynomial of *L/A*. Thus, we define a correction factor,*η*, as a linear function of *L*/*A*.


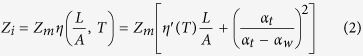


Eq. 2 explicitly indicates that the linear extrapolation of *Z*_*m*_ to *L/A* = 0 gives *Z*_*i*_.

The slope of *η*, *η*′ = *dη/d*(*L*/*A*), depends on the temperature-dependent physical properties, especially on *K*_*w*_/2 *k*_*t*_. In Eq. 1, if 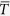
−*T*_*0*_ < 1 K and *I*^*2*^ *ρ*~μ Wcm, the second term in the square bracket is about two orders of magnitude smaller than the third term (see Fig. S4 in supplementary material). Eq. 1 can be further simplified. If Cu electric wires (*α*_*Cu*_ = 1.8–2.8 μV/K at 300 K ≤ *T* ≤ 500 K) are used for a sample with *α*_*t*_~200 μV/K, [*α*_*t*_/(*α*_*t*_−*α*_*w*_)]^2^ becomes ~1. Thus, for the Harman measurement using a highly efficient TE material with a good radiation shield, a simplified form of *η* exists as


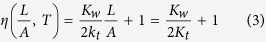


Eq. 3 implies that the Harman method error due to the PTEs can be small when *K*_*t*_ overwhelms *K*_*w*_. However, practically it is difficult to achieve *K*_*t*_ ≫ *K*_*w*_, since too thin electric wires would result in Joule heating and more difficulty in the electric wire welding on the sample.

To determine *k*_*t*_ and *K*_*w*_, Eq. 1 is modified by substituting *Z*_*i*_ with *α*_*t*_^*2*^/*ρ*_*t*_*k*_*t*_.


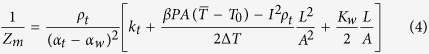


If 1/*Z*_*m*_ is available as a function of *L/A*, the measured data can be fitted to Eq. 4. For this fitting, *α*_*t*_ should be measured independently, and *ρ*_*t*_ and Δ*T* should be extracted from *V*_*ac*_ and *V*_*dc*_, respectively. If 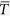
~*T*_*0*_, then only unknowns in Eq. 4 are *k*_*t*_ and *K*_*w*_. Fortunately, *k*_*t*_ is a factor for the y-axis intercept and *K*_*w*_ is a factor for the slope in Eq. 4. The fitted *k*_*t*_ is used to calculate *Z*_*i*_.

### Model for TEM measurement

Figure 1b shows the schematic for the TEM measurement. Unlike the material measurement configuration, the electric wires are at only one side of the sample. Based on the one-dimensional (1D) heat transfer model for a TEM^15^, an energy balance at the hot-side substrate is as following.


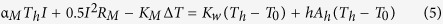


where *h* is the convective heat transfer coefficient. The subscripts *M* and *h* denote the TEM and the hot-side substrate, respectively. Eq. 5 shows that the net heat flow at the hot-side substrate is balanced with the heat dissipation via the electric wires and air convection. To estimate Δ*T*, the right-hand side of Eq.5 is simply modified as *K*_*e*_Δ*T* where *K*_*e*_ combines *K*_*w*_, *hA*, and the ratio between Δ*T* and *T*_*h*_−*T*_*0*_. Then, Δ*T* is expressed as


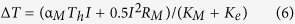


Measured *α* and *k* of the materials estimated *α*_*M*_ and *K*_*M*_. *R*_*M*_ was acquired via the measurement of *V*_*ac*_.

Although the 1D heat transfer model effectively captures the relevant thermal phenomena, it does not account for the contribution of the substrates. Intuitively, the thermal conductance along the substrate would affect the temperature distributions of the substrates and TE materials. Thus, we developed a three-dimensional TE finite element model (FEM) using a commercial software package (COMSOL Multiphysics) for the TEM measurement. The model calculates the temperature and electrical field distributions when DC is applied. To simulate the heat flow via electric wires, one of the substrates is subject to a uniform heat flux which is equal to *K*_*w*_(*T*−*T*_*0*_)/*A*, where *K*_*w*_ is obtainable by fitting the calculated data to the experimental data. As a thermal boundary condition, the other substrate temperature is set to *T*_*0*_. Since the FEM simulates the measurement under vacuum condition, all other surface area of a TEM is thermally insulated. As an electrical boundary condition, one of the TEM electrode is electrically grounded, while the other TEM electrode serves as a DC current source. To electrically insulate the substrate, the electrical conductivity and Seebeck coefficient of the substrates are set to zero. Other details of FEM are included in the supplementary information.

## Results and Discussion I: Material measurement

Through measuring *Z* as a function of the sample size, we observe the PTEs both in the material and TEM measurements. By comparing the model with the measured data, we aim to understand how the PTEs are determined and corrected.

**Figure 2:**
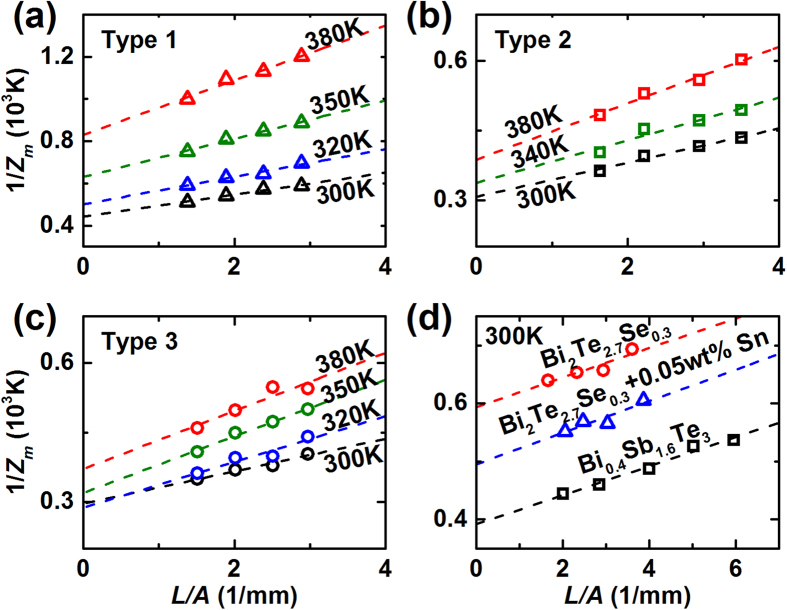
Measured inverse figure-of-merit (1/*Z*_*m*_) for TE material samples. For the samples prepared by hot extrusion (**a**–**c**), 1/*Z*_*m*_ were measured in the temperature range of 300–380 K. For the samples prepared by SPS and hot press, 1/*Z*_*m*_ were measured only at 300 K.

Figure 2 shows 1/*Z*_*m*_ of the test materials as a function of *L*/*A*. As Eq. 2 predicts, 1/*Z*_*m*_ shows a linear dependence on *L*/*A* for all the temperatures and material types. The y-axis intercepts correspond to 1/*Z*_*i*_, and are used to determine *k*_*t*_. Based on Eq. 4, the slopes are related to *K*_*w*_, hence the slope would change if different types of electric wires or wiring processes are employed. Here, the samples seem to possess comparable *ρ*_*t*_*K*_*w*_/(*α*_*t*_−*α*_*w*_)^2^ each other such that the slopes are similar when the measuring temperatures are identical.

**Figure 3:**
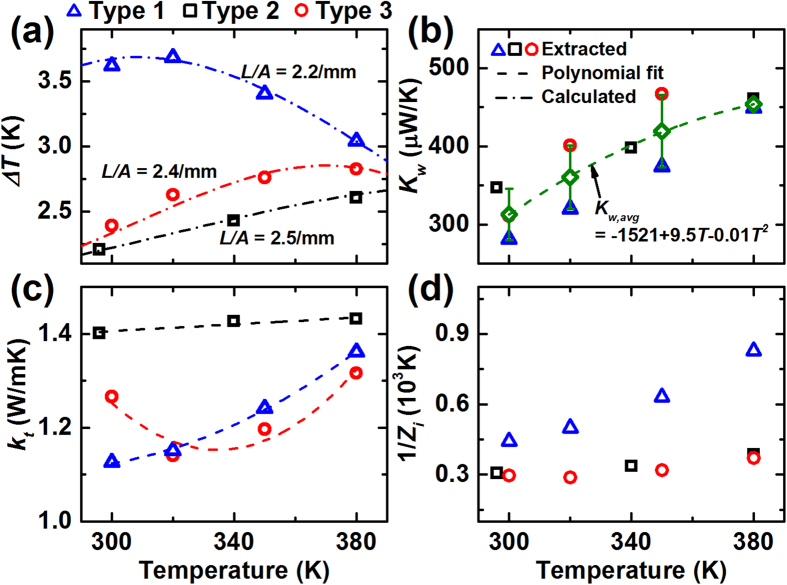
TE properties for material samples extracted by model fits. (**a**) Temperature difference between the hot and cold sides (*ΔT*), (**b**) thermal conductance through the electric wires (*K*_*w*_), (**c**) thermal conductivity, (**d**) inverse of the corrected figure-of-merit.

By fitting Eq. 4 to the experimental data, TE properties and *K*_*w*_ are uncovered. For this fitting, Δ*T* is extracted from *V*_*dc*_ and *V*_*ac*_ as shown in Fig. 3a. *K*_*w*_ and *k*_*t*_ are simultaneously fitted by the least square method. Figure 3b shows that *K*_*w*_ for various materials are similar with a deviation, *σ*, ≤ ± 40 μW/m. The deviation of *K*_*w*_ would result from the differences of the electric wire length and inconsistent thermal contact resistance at the wire-sample interfaces. An average value of *K*_*w*_ (*K*_*w,avg*_) for the samples could be fitted to a second order polynomial in the given temperature range. Figure 3c shows the fitted *k*_*t*_ that turn out to be comparable to the reported values of the similar materials^16^,^17^. With these *k*_*t*_, *Z*_*i*_ is obtainable as shown in Fig. 3d, and Δ*T* can also be calculated as shown in Fig. 3a. The calculated Δ*T* are close to the experimentally extracted Δ*T*, indicating that fitted *K*_*w*_ and *k*_*t*_ are reasonable values.

**Figure 4:**
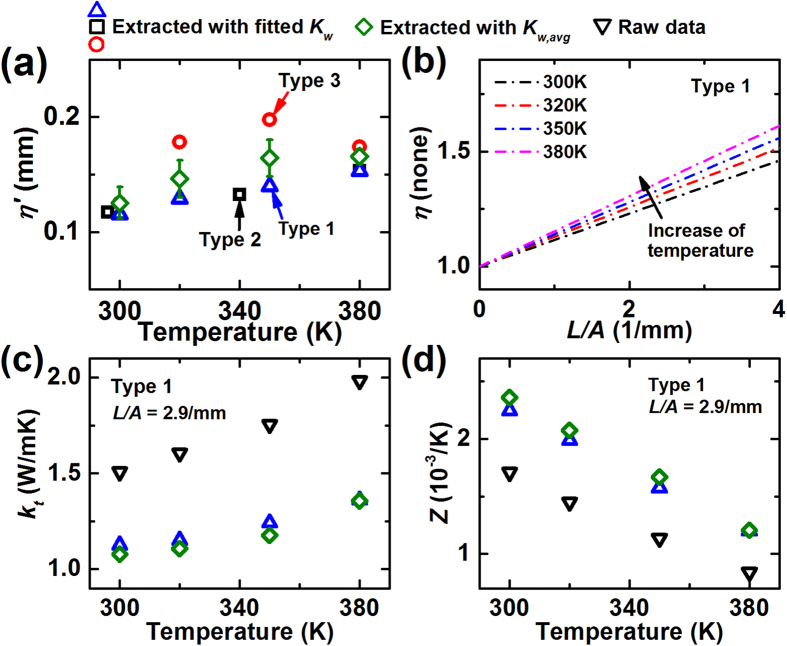
(**a**) Correction factor slopes for material samples acquired at discrete temperature points. (**b**) Correction factor, (**c**) thermal conductivity, (**d**) *Z* of type 1. (**c**,**d**) To observe the influence of the uncertainty of *K*_*w*_, the TE properties were extracted using two *K*_*w*_s: (1) *K*_*w*_ that was particularly fitted for type 1, and (2) *K*_*w,avg*_.

Assuming that the PTEs are continuous functions of the temperature and the sample geometry, the correction factor, *η*, for an arbitrary *L*/*A* and *T* is predictable with discrete values of *η*. Figure 4a and b show *η*′ and *η* for the test materials. By fitting the discrete values of *η*′ to a polynomial, *η*′ could be interpolated within the relevant temperature range for the particular type of material. Interestingly, type 1 and 2 show similar *η*′, while type 3 exhibits comparatively large *η*′. The deviation of *η*′ between the samples is due to the difference of the ratio of *K*_*w*_ to *k*_*t*_ as Eq. 3 indicates. Since *k*_*t*_ of the samples are similar, here *K*_*w*_ is a key factor for *η*′. If *η*′ is estimated with *K*_*w,avg*_, the deviation of *η*′ becomes much smaller (<10%), implying that the uncertainty of *K*_*w*_ is critical for *η*′.

Figure 4c and d show how the uncertainty of *K*_*w*_ affects the uncertainties of estimated *k*_*t*_ and *Z*, especially for type 1 with *L*/*A* = 2.9/mm. To estimate the influence of the *K*_*w*_ uncertainty, we corrected the raw data using two different *K*_*w*_: (1) *K*_*w*_ that were particularly fitted for type 1 (*K*_*w,1*_), and (2) *K*_*w,avg*_. Although *K*_*w,avg*_ is ~10% larger than *K*_*w,1*_, *k*_*t*_ and *Z* corrected by the two *K*_*w*_ exhibit ~5% deviations to each other. Therefore, fortunately, the uncertainty of *K*_*w*_ seems to give reduced impact on the uncertainties of *k*_*t*_ and *Z*. To further reduce the uncertainties for correcting *k*_*t*_ and *Z*, a reliable and consistent electrical contact should be necessary. Another approach would be to measure a sample with small *L/A* where *η* is small such that the absolute influence of the *K*_*w*_ uncertainty becomes negligible.

## Results and Discussion II: TEM measurement

**Figure 5:**
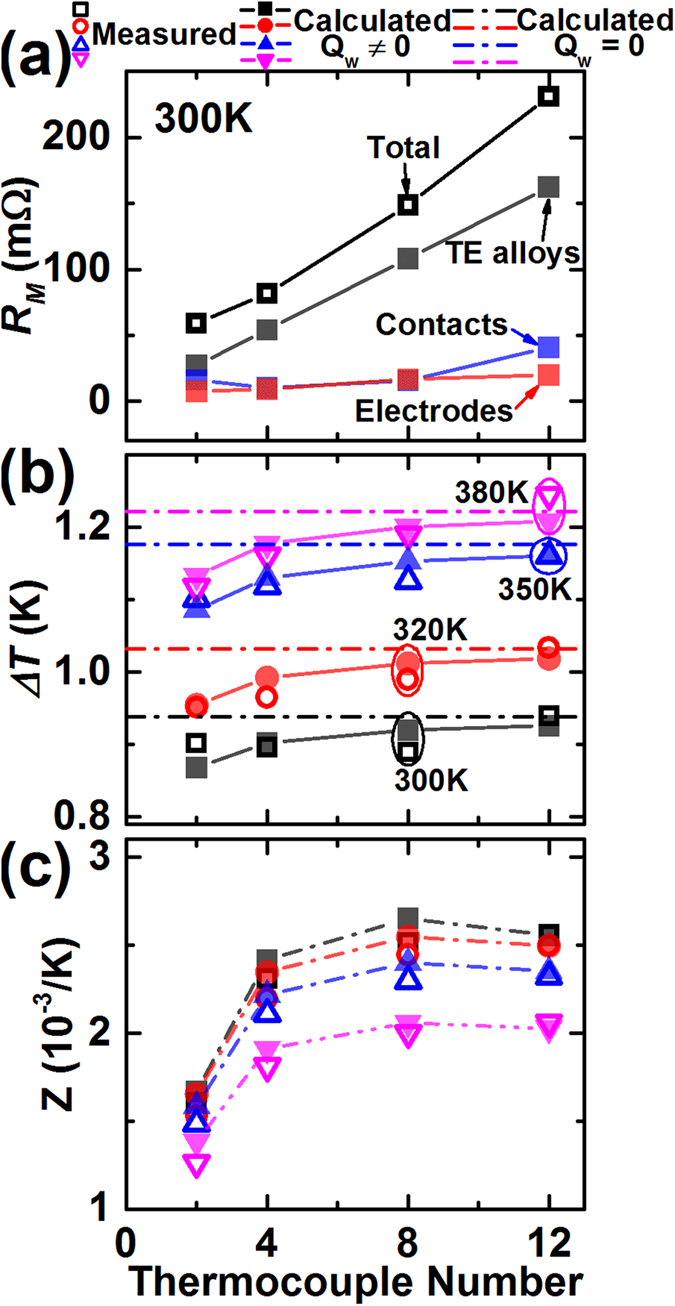
TE properties of TEMs as a function of the TEM size, equivalently thermocouple number. (**a**) Electrical resistance, (**b**) temperature difference between the hot and cold sides (*ΔT*), (**c**) *Z*.

Figure 5 shows the TE properties of the TEMs as a function of the thermocouple number (*n*). Measured electrical resistance of the TEMs (*R*_*M*_) increases with *n* which is the sum of the resistances of TE materials (*R*_*TE*_), electrodes (*R*_*elec*_), and contact resistance at the interfaces (*R*_*cont*_). Here, *R*_*cont*_ was estimated by *R*_*M*_−*R*_*TE*_−*R*_*elec*_. As *n* becomes small, *R*_*elec*_ and *R*_*cont*_ decrease without exhibiting complete linear-trends. The nonlinearity arises from the fact that the total length of the TEM electrodes does not increase linearly with *n* and the soldering quality would vary without an automated TEM fabrication. In our case, *R*_*cont*_/*n* is ~2 mΩ. As *Z* is determined by *α*_*M*_Δ*T*/*IR*_*M*_, the relative magnitude of *R*_*elec*_ + *R*_*cont*_ should be small for *Z* to be large.

Heat flow through the electric wires (*Q*_*w*_) affects the temperature gradient within the TEM as well as the estimated *Z*. As the impact of *Q*_*w*_ is not the intrinsic property of the TEM, the measured *Z* should be calibrated by a factor of (*K*_*M*_ + *K*_*e*_)/*K*_*M*_, especially for a small-sized TEM. Figure 5b shows that experimentally extracted Δ*T* slightly reduces as *n* decreases. Eq. 6 predicts well the dependence of Δ*T* on *n* employing a single value of *K*_*e*_ (=500 μW/K). If *Q*_*w*_ is 0 (ideal condition which is equivalent to *K*_*e*_ = 0), then Δ*T* should be a constant regardless of *n*. When *Q*_*w*_ is not 0 (realistic condition) and a TEM has small *n*, however, *K*_*M*_is small (e.g. *K*_*M*_~7 mW/K for *n* = 2) such that the influence of *Q*_*w*_ becomes important. Thus, if comparing the measured *Z* (*Z*_*m*_) and the calculated *Z* with *Q*_*w*_ = 0 (*Z*_*adiabatic*_), they differ 5–10% for small TEMs. On the other hand, for large TEMs (*K*_*M*_/*K*_*w*_ > 50), the influence of *Q*_*w*_ becomes small, hence the difference between *Z*_*m*_ and *Z*_*adiabatic*_ reduces to less than 1%.

**Figure 6:**
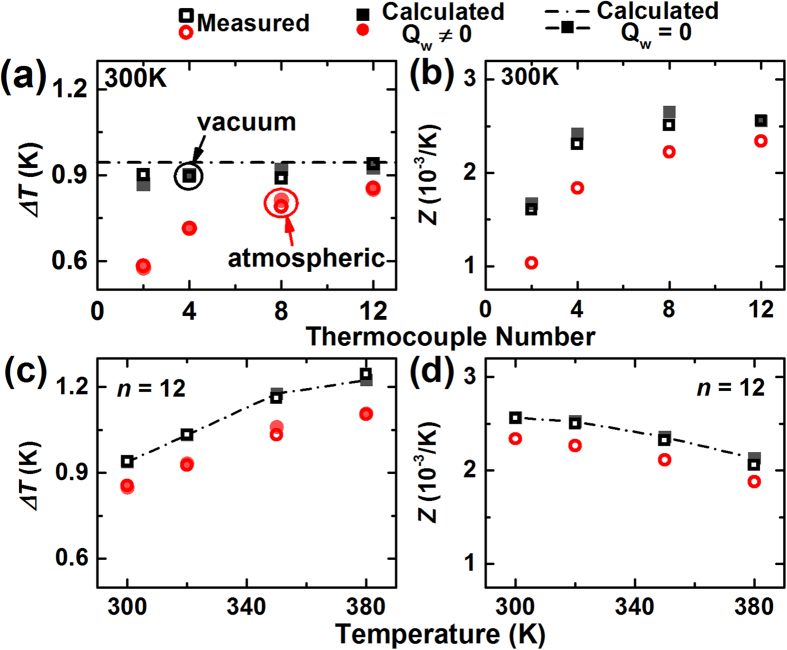
Comparison of the TEM measurements under vacuum (square) and atmospheric condition (circle). At 300 K, (**a**) *ΔT*, and (**b**) *Z* were obtained as a function of the TEM size (*n*). For the TEM with *n* = 12, (**c**) *ΔT*, and (**d**) *Z* were acquired in the temperature range of 300–380 K.

For the TEM measurement in air, the convective heat transfer (*Q*_*conv*_) becomes important, and causes seriously underestimated *Z*. Figure 6a and b show the measured Δ*T* and *Z* of the TEMs at 300 K. The data measured in air is 10–35% lower than the data acquired in vacuum, although the deviation decreases with larger *n*. The contribution of *Q*_*conv*_ could be estimated by calculating Δ*T*. With *K*_*e*_ of 4 mW/K, Δ*T* measured in air were fitted. With this *K*_*e*_ and the TEM substrate area, the convective heat transfer coefficient for this TEM measurement was estimated as ~20 W/m^2^K. The deviation between the data measured in vacuum and air slightly increases as the measuring temperature increases. Figure 6c and d show Δ*T* and *Z* of the TEMs with *n* = 12. Although the influence of *Q*_*w*_ is not great, the effect of *Q*_*conv*_ is evident by causing an error of ~10%.

**Figure 7:**
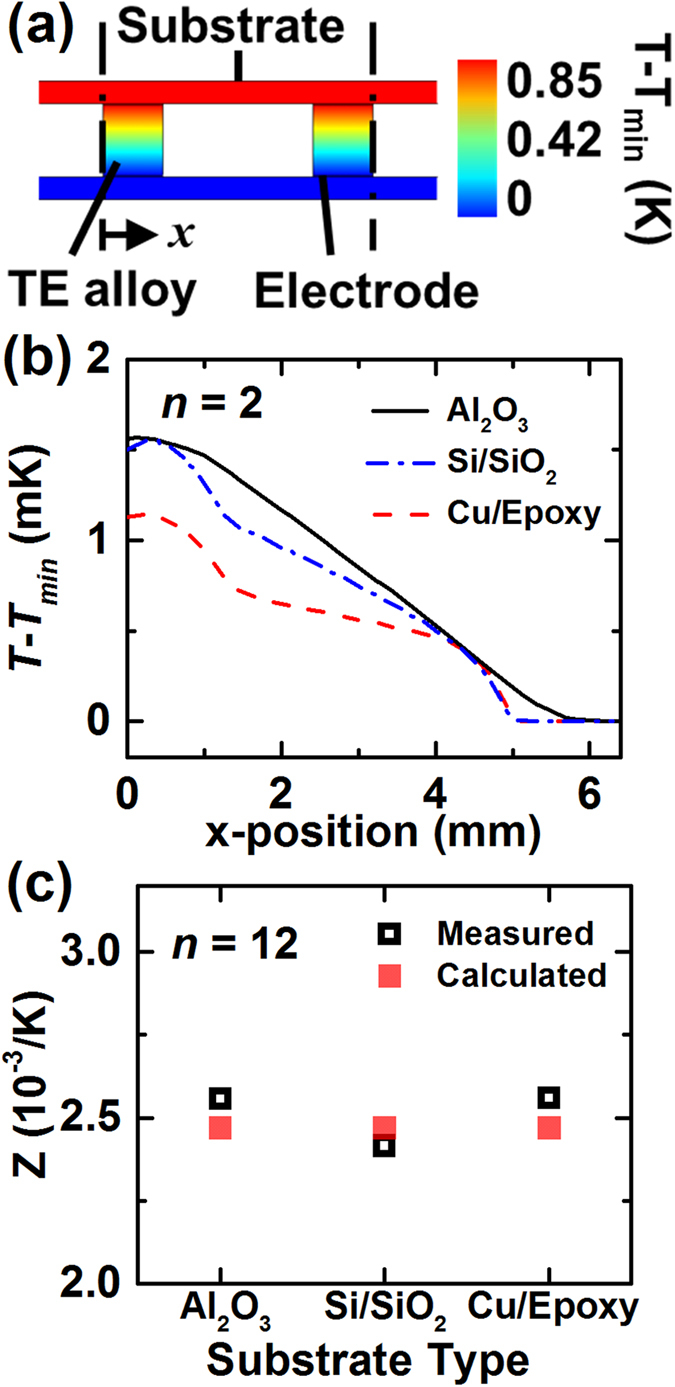
Substrate effect in the TEM measurement. (**a**) Temperature distribution of a TEM with Al_2_O_3_ substrates calculated by the FEM. For the TEMs with different types of substrates, (**b**) predicted temperature profiles along the bottom TEM substrate and, (**c**) *Z* were obtained at *T*_*0*_ = 300 K.

Another concern in the TEM measurement is the substrate effect. Intuitively, highly conductive substrate is likely to increase *Q*_*w*_ as it spreads heat well. Figure 7a shows the temperature distribution calculated by the FEM for a TEM with *n* = 2 and alumina substrates. Likewise, the temperature profiles along the substrates were calculated for other types of substrate as shown in Fig. 7b. The calculated result shows that the difference of the temperature profiles among the substrate materials is ≤1 mK. The substrate type seems not make meaningful difference, as any substrate type used here has sufficiently large in-plane thermal conductance. *K*_*w*_ for the calculation was obtained by fitting *Z* to the measured data as shown in Fig. 7b. *K*_*w*_ was in the range of 100–200 μW/K. Figure 7c shows the measured and calculated *Z* of the TEMs where the deviation of the measured data is mostly due to the variations of the TE material properties. However, it should be noted that the substrate effect might become important if the in-plane thermal resistance of the substrate (*R*_*th,sub*_) is relatively large as compared to the total thermal resistance of the TEM (*R*_*th,TEM*_). For the TEMs used in this study, *R*_*th,sub*_ is ~6–65% of *R*_*th,TEM*_. If the substrate thickness reduces to few tens of μm or the TE leg length reduces an order of magnitude, *R*_*th,sub*_/*R*_*th,TEM*_ will become much larger than unity, and the heat transfer across the substrate would affect *Q*_*w*_ and the Harman measurement.

## Conclusions

The effect of the PTEs in the Harman measurement was investigated theoretically and experimentally. For the material measurement, the dependence of 1/*Z*_*m*_ on *L*/*A* becomes linear if the radiative heat transfer and Joule heating are constricted well by employing a radiation shield and small current. By fitting the model to 1/*Z*_*m*_ at discrete *L*/*A*s and *Ts*, a continuous correction factor is predictable, which is useful to correct *Z* and thermal conductivity for a certain *L*/*A* and *T*. However, the uncertainty of the correction depends on the uncertainty of *K*_*w*_, which is difficult to accurately estimate. For the TEM measurement, the heat flow through electric wires and air are also important. Especially, when the thermal conductance between the TEM and the environment becomes relatively large (*K*_*e*_/*K*_*M*_ > 10%), the measurement error could be significant (10–35%). Thus, an adiabatic condition and a proper correction are critical to characterize the intrinsic TE properties of the TEM. This work will be helpful to characterizing the TE materials and modules, and understanding the sample-size dependent data.

## Additional Information

**How to cite this article**: Roh, I.-J. *et al*. Harman Measurements for Thermoelectric Materials and Modules under Non-Adiabatic Conditions. *Sci. Rep.* **6**, 39131; doi: 10.1038/srep39131 (2016).

**Publisher's note:** Springer Nature remains neutral with regard to jurisdictional claims in published maps and institutional affiliations.

## Supplementary Material

Supplementary Information

## Figures and Tables

**Table 1:** Properties at 300 K and dimensions of the test materials and the materials used in TEMs.

	Type 1	Type 2	Type 3	TEM legs	
				*p*-type	*n*-type
Composition	Bi_2_Te_2.64_Se_0.36_	Bi_2_Te_2.7_Se_0.3_	Bi_0.45_Sb_1.55_Te_3_	Bi_2−*x*_Sb_*x*_Te_3_	Bi_2_Te_3−*x*_Se_*x*_
*α*_*t*_ (μV/K)	−290	−229	207	196	−198
*ρ*_*t*_ (mΩcm)	3.3	1.2	1.0	0.9	0.8
*k*_*t*_ (W/mK)	1.1	1.4	1.3	1.3	1.8
*K*_*w*_ (μW/K)	281	347	311	—	—
*Z*_*i*_ (10^−3^/K)	2.25	3.23	3.41	3.26	2.74
Area, *A* (mm^2^)	7.1	6.6	6.8	2.0	
Length, *l* (mm)	9.8–20.4	10.9–23.3	10.3–20.3	1.6	

**Table 2:** Properties at 300 K and details of TEMs.

Number of thermocouples	2	4	8	12		
*α*_*M*_ (μV/K)	780	1560	3120	4680		
*R*_*M*_ (mΩ)	58	81	149	231	219	239
*K*_*M*_ (m*W/K)*	7	13	26	37	39	38
*Z* (10^−3^/K)	1.61	2.31	2.51	2.56	2.56	2.42
Area (mm^2^)	10 × 14.5				10 × 17	
Substrate material	Cu (530 μm)				Al_2_O_3_ (530 μm)	Si (530 μm)
Insulating material	Epoxy (30 μm)				—	SiO_2_ (1 μm)
Electrode material	Cu (35 μm)					
