# Supplementary material for: Harman Measurements for Thermoelectric Materials and Modules under Non-Adiabatic Conditions
Source: Sci Rep. 2016 Dec 14;6:39131. doi: 10.1038/srep39131 (PMC5155428; doi:10.1038/srep39131)
Supplement: Supplementary Information [file srep39131-s1.pdf]

Supplementary Information

Harman Measurements for Thermoelectric Materials and Modules under Non-Adiabatic Conditions

Im-Jun Roh[1], Yun Goo Lee[1,2], Min-Su Kang[1,2], Jae-Uk Lee[1], Seung-Hyub Baek[1,3], Seong Keun Kim[1], Byeong-Kwon Ju[2], Dow-Bin Hyun[1], Jin-Sang Kim[1] & Beomjin Kwon[1]

[1]*Center for Electronic Materials, Korea Institute of Science and Technology (KIST), Seoul, Republic of Korea 02792*

[2]*Display and Nanosystem Laboratory, College of Engineering, Korea University, Seoul, Republic of Korea 02841*

[3]*Department of Nanomaterials Science and Technology, Korea University of Science and Technology, Daejeon, Republic of Korea 34113*

1. Temperature dependent TE properties for the test materials

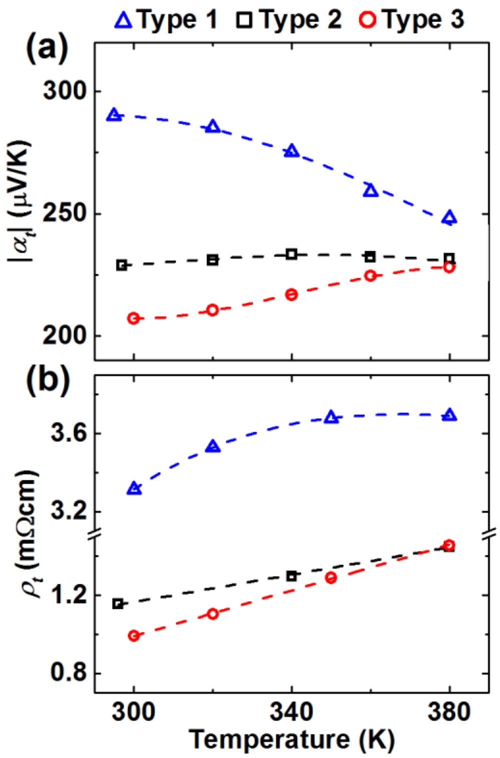

Supplementary Figure S1. Measured (a) Seebeck coefficient and (b) electrical resistivity of the test materials in the temperature range of 300 – 380 K.

2. Temperature dependent TE properties for the materials used for TEMs

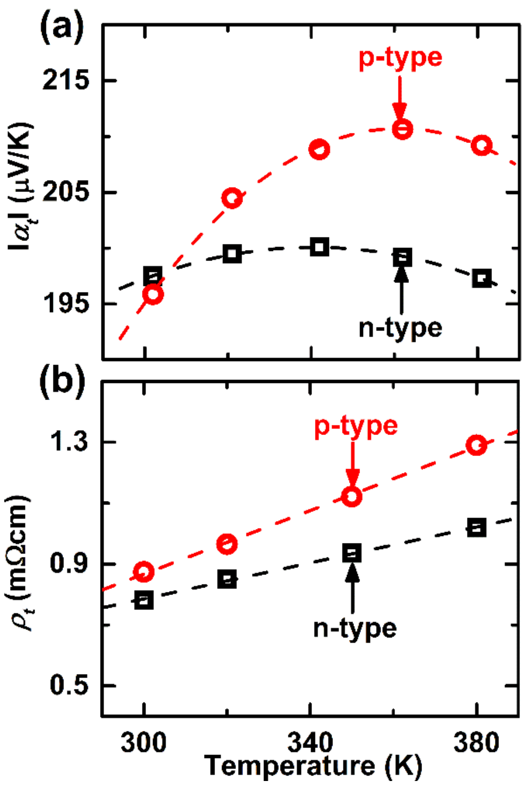

Supplementary Figure S2. Measured (a) Seebeck coefficient and (b) electrical resistivity of the materials used for TEDs in the temperature range of 300 – 380 K.

3. Derivation of ΔT across the material sample during the Harman measurement

Assuming the sample has one-dimensional temperature variation without convective heat transfer, the steady state energy balance becomes

$$k_t A \frac{d^2T}{dx^2} + I^2 \frac{\rho_t}{A} - \beta P(T - T_0) = 0 \tag{S1}$$

where x is the distance from one end of the sample. Then, a solution for Eq. S1 is

$$T(x) - T_0 = c_1 e^{\sqrt{\beta P/k_t A}x} + c_2 e^{-\sqrt{\beta P/k_t A}x} + \frac{I^2 \rho_t}{\beta P A} \tag{S2}$$

where c_1 and c_2 are constant coefficients determined by two boundary conditions. The boundary conditions are the energy balance at $x = 0$ and L. Thus,

$$T(0) - T_0 = c_1 + c_2 + \frac{I^2 \rho_t}{\beta P A} \tag{S3}$$

$$T(L) - T_0 = c_1 e^{\sqrt{\beta P/k_t A}L} + c_2 e^{-\sqrt{\beta P/k_t A}L} + \frac{I^2 \rho_t}{\beta P A} \tag{S4}$$

$$\Delta T = c_1 \left(e^{\sqrt{\beta P/k_t A}L} - 1\right) + c_2 \left(e^{-\sqrt{\beta P/k_t A}L} - 1\right) \tag{S5}$$

Assuming the sample is hot at $x = 0$ and is cold at $x = L$, $T(0)$ and $T(L)$ can be denoted as T_h and T_c, respectively. An energy balance at $x = 0$ is

$$(\alpha_t - \alpha_w) I T_h = -kA \left.\frac{dT}{dx}\right)_{x=0} + K_w (T_h - T_0) \tag{S6}$$

And an energy balance at $x = L$ is

$$(\alpha_t - \alpha_w) I T_c = -kA \left.\frac{dT}{dx}\right)_{x=L} + K_w (T_0 - T_c) \quad \text{(S7)}$$

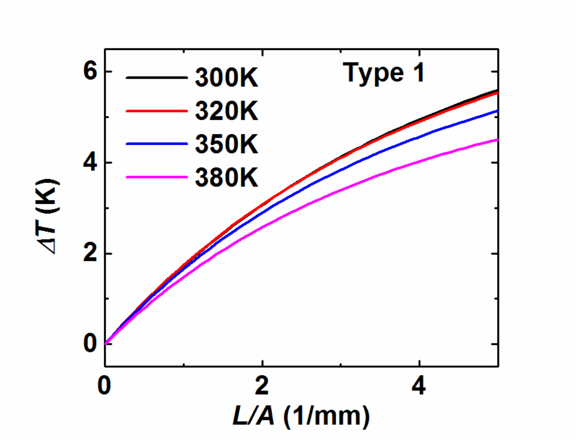

Supplementary Figure S3. Calculated ΔT of type 1 as a function of L/A in the temperature range of 300 – 380 K.

4. Comparison of the components included in the correction factor

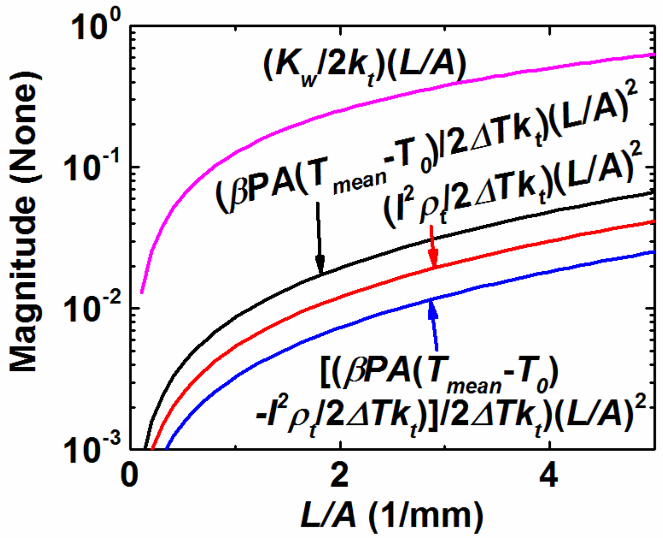

Supplementary Figure S4. Calculated components of the correction factor for type 1 as a function of *L/A* at 300K. The calculation assumes that T_{mean}-T_0 = 0.5K and β = 1.

5. Information for FEM

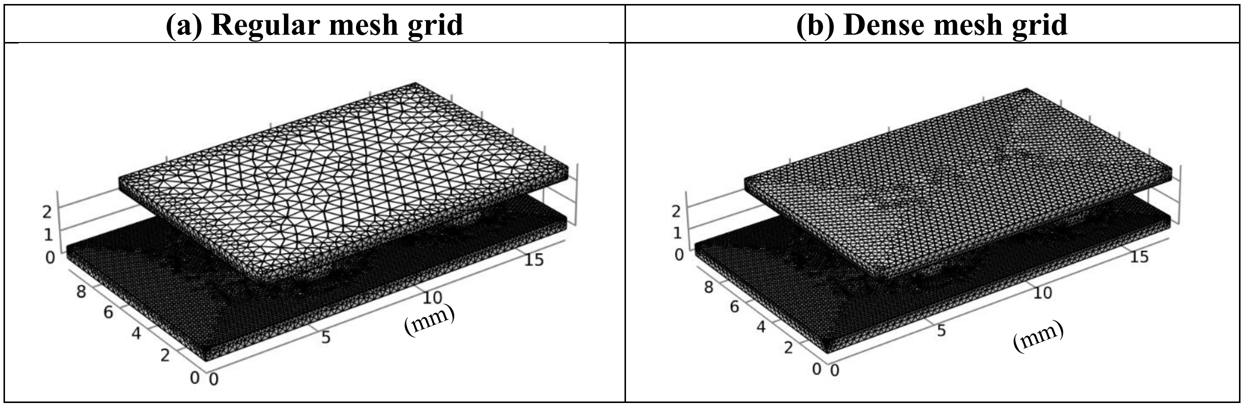

Supplementary Figure S5. Meshed structures of the three-dimensional FEM of a TEM with (a) regular mesh grid, (b) dense mesh grid.

Supplementary Table S1. Comparison between regular and dense mesh grids

	Regular mesh grid	Dense mesh grid
Maximum size of mesh (D_{max})	0.935 mm	0.34 mm
Substrate length/D_{max}	10.48(9.8 mm/0.935 mm)	28.82(9.8 mm/0.34 mm)
Minimum size of mesh (D_{min})	0.035 mm	0.0034 mm
Electrode thickness/D_{min}	1(0.035 mm/0.035 mm)	10.29(0.035 mm/0.0034 mm)
V_{DC} (mV)	1.57	
Z (10^{-3}/K)	2.48	

Supplementary Table S2. Thermal conductivities of the substrate materials used in FEM

	Cu	Epoxy resin mixed with boron particles	Al_2O_3	Si	SiO_2
k (W/mK)	401	6	36	148	1.38

To select an appropriate size of mesh, we calculated V_{DC} and Z by varying the mesh size. To ensure the FEM produces a physically realistic and stable solution, first, dense mesh grid was employed where more than 10 mesh points exists even within the thinnest feature (electrode layer). To reduce the computation cost, we increased the mesh size to the regular mesh grid level, and compared with the result from FEM with the dense mesh grid (Table S1).

Table S2 shows the thermal conductivities of the substrate materials used in FEM. The

thermal conductivities of Cu, Al_2O_3, Si, and SiO_2 are from literature (Reference: Incropera, F. P. and Dewitt D. P. Fundamentals of heat and mass transfer, 7th ed., John Wiley & Sons, Inc., 2011) and the thermal conductivity of epoxy composite is a nominal value from the manufacturer. As addressed in the main article, Seebeck coefficient and electrical conductivity of the substrate materials were set to zero for an electrical insulation. The physical properties for TE legs are shown in Table 1.
